# Deposition of an Addressable Molecular Spin Qubit with Built-In Decoupling Structure

**DOI:** 10.1021/jacs.6c01396

**Published:** 2026-05-07

**Authors:** Niccolò Giaconi, Leonardo Tacconi, Matteo Briganti, Alessio Nicolini, Olga Mironova, Marta Albanesi, Julie Lion, Fabio Santanni, Edwige Otero, Philippe Ohresser, Giulia Serrano, Lorenzo Poggini, Andrea Cornia, Matteo Mannini

**Affiliations:** † Department of Chemistry “Ugo Schiff” (DICUS) and INSTM Research Unit, 9300 University of Florence, Via della Lastruccia 3-13, Sesto Fiorentino 50019, Italy; ‡ Department of Chemical and Geological Sciences and INSTM Research Unit, 124246 University of Modena and Reggio Emilia, Via G. Campi 103, Modena 41125, Italy; § Synchrotron-SOLEIL, L’Orme des Mérisiers, Saint-Aubin 91190, France; ∥ Department of Industrial Engineering (DIEF) and INSTM Research Unit, University of Florence, Via Santa Marta 3, Florence 50139, Italy; ⊥ Institute for Chemistry of Organo-Metallic Compounds (ICCOM-CNR), Via Madonna del Piano, Sesto Fiorentino 50019, Italy

## Abstract

The integration of molecular spin qubits in the next generation of quantum devices requires magnetic centers that can be individually addressed while remaining decoupled from the substrate. Envisioning this future perspective here, we introduce a heterobimetallic molecular design strategy that integrates a paramagnetic vanadyl spin center with a built-in inorganic decoupling unit within a single coordination complex, overcoming conventional approaches that rely on inorganic buffer layers such as MgO and thereby limit versatility and scalability. The lantern complex [PtVO­(SOCPh)_4_] (PtVO) embeds a VO^2+^ qubit spatially shielded by a square-planar PtS_4_ moiety eliminating the need for external decoupling layers. A submonolayer of PtVO was successfully deposited on a highly oriented pyrolytic graphite substrate via electrospray deposition, yielding a chemically intact and well-defined molecular interface. Combining element and polarization-resolved synchrotron spectroscopies, supported by density functional theory calculations, demonstrates that the vanadyl center remains magnetically isolated at the submonolayer limit. Polarization- and angular-dependent X-ray absorption spectroscopy, flanked by multiplet ligand field theory simulations, provided detailed insight into the adsorption geometry and the electronic structure of PtVO upon deposition. Angular-dependent X-ray magnetic circular dichroism further reveals how the molecular coordination geometry governs the orbital contributions and magnetic anisotropy of square-pyramidal vanadyl systems. These results establish a built-in molecular decoupling system as a viable chemical principle for the scalable integration of addressable molecular spin qubits on low-dimensional materials, paving the way to new routes toward surface-based quantum architectures.

## Graphical abstract

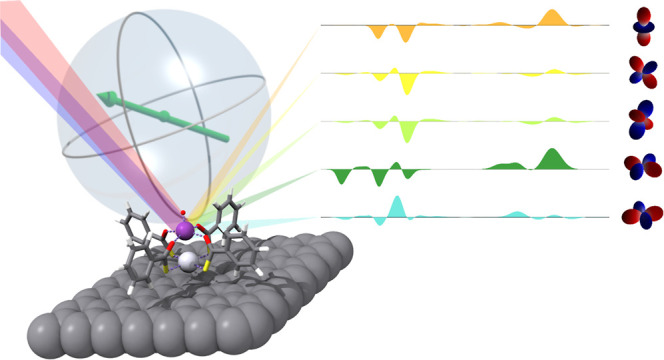

## Introduction

Single spin addressing is one of the main challenges in integrating molecular qubits in the next generation of quantum devices. This would require organizing such units into ordered 2D arrays and, simultaneously, achieving subnanometric control of their spin states. Over the past few years, scanning tunnelling microscopy coupled with electron spin resonance has enabled the investigation of the quantum coherence of single magnetic molecules and single adatoms, suggesting their use as the ultimate level of miniaturization and, more recently, demonstrating the capability of encoding quantum logical operations in multiqubit architectures.^1,2^

With their flat structure and high symmetry, metal porphyrins and phthalocyanines are especially prone to self-assemble into ordered monolayers and are the most widely investigated spin qubits on surfaces.^3,4^ In particular, vanadyl porphyrins and phthalocyanines exhibit appreciable coherence times above 1 μs^4–10^ and enable both qubit and qudit implementations thanks to the *S* = 1/2 and *I* = 7/2 spin states.^11^ Unfortunately, the metal orbitals in these flat systems can hybridize with the underlying surface, leading to detrimental consequences for quantum properties.^12–14^ To reduce these effects, the investigation of such planar systems requires the introduction of a suitable decoupling layer. Several materials have been tested, including NaCl,^15,16^ Al_2_O_3_,^17^ and, most notably, MgO.^18,19^ The latter has demonstrated the highest efficiency in suppressing interactions with conductive electrons and enhancing the quantum coherence of the target systems. One drawback of MgO is that it can exhibit a high density of defects and preferential island growth,^20^ which are detrimental to scalability. Furthermore, its oxygen atoms can significantly alter the crystal field splitting of metal orbitals, thereby affecting magnetic and quantum properties.

A possible way to circumvent these limitations is to design molecules with a built-in insulating atomic layer. In this way, the molecular electronic states carrying spin information are effectively decoupled from the electronic bands of the substrate, while the magnetic and quantum properties of the spin center are expected to be maintained after adsorption. Nonplanar molecular systems like lantern (or paddlewheel) complexes provide a versatile platform for this purpose. With their simple and robust structure, consisting of two metal centers bridged by multiple ligands, lantern complexes have found extensive applications in several research areas, including catalysis, magnetism, and molecular electronics.^21^ While the most common lantern complexes feature {O,O}-donor carboxylate ligands and homometallic magnetic centers,^22^ the recent introduction of monothiocarboxylate ligands has provided access to a wide range of heterobimetallic architectures containing an O-bonded transition metal ion and an S-bonded group 10 metal ion (M) with no unpaired electrons, like Pt^2+^ or Pd^2+^.^23^ The rare available examples of vanadyl derivatives of this type hold promise as molecular spin qubits featuring coherence times longer than those observed for the equivalent porphyrins and phthalocyanines.^24,25^ In their structure, the metal ion M has a square planar coordination geometry, which imposes a 4-fold molecular symmetry (Figure 1a). Upon deposition on a surface, the flat and electronically soft MS_4_ moiety can act as a preferential surface-binding site, dictating a standing orientation of molecules and, at the same time, serving as a built-in decoupling layer for the vanadyl spin center.

**Figure 1:**
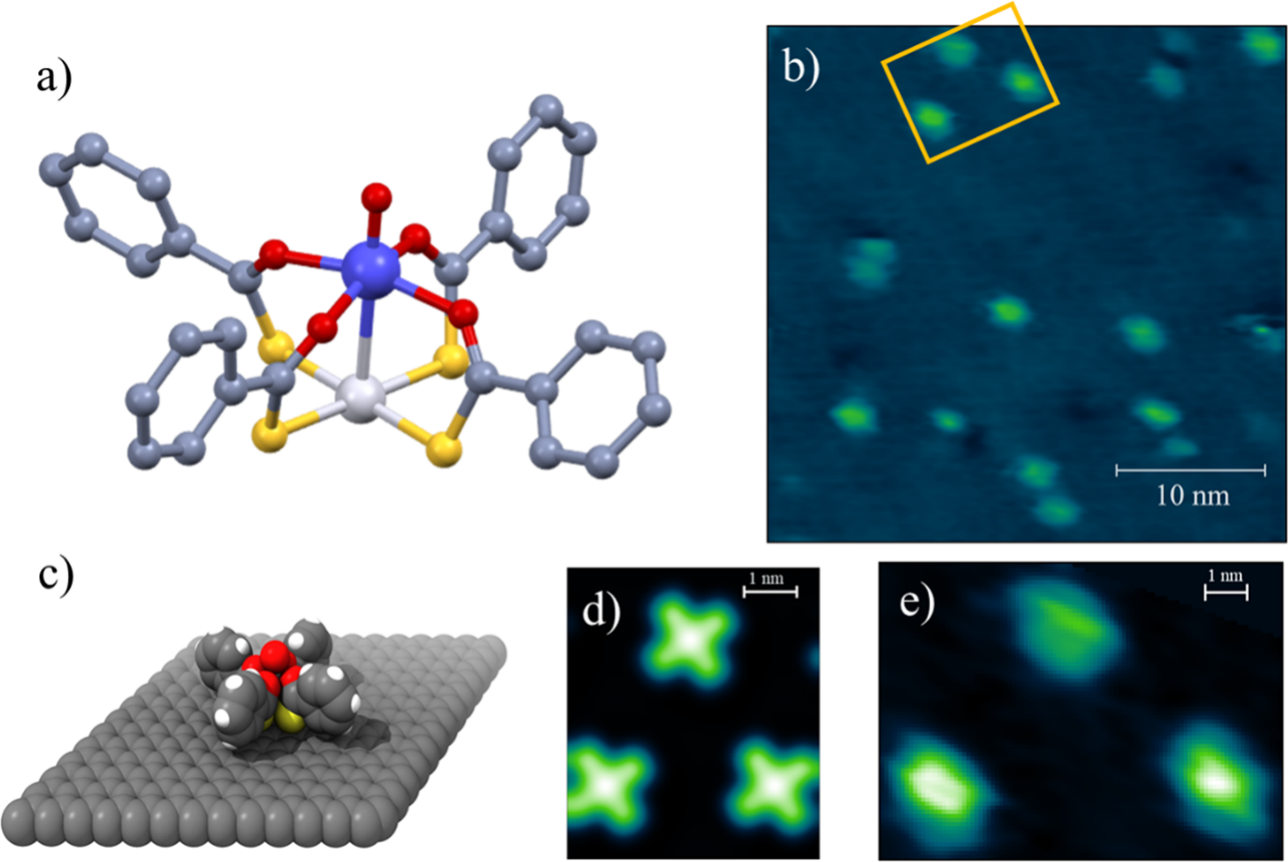
(a) Structure of the **PtVO** molecule omitting hydrogen atoms. Atom color code: C, dark grey; O, red; S, yellow; Pt, light grey; V, indigo. (b) STM image of **PtVO** deposited on HOPG recorded at 35 K (35 × 35 nm^2^, *V* = 2.5 V, *I*_*t*_ = 2 pA). (c) Most stable configuration of **PtVO** on graphene at the pDFT level of theory. (d) Corresponding simulated STM image of **PtVO** in standing configuration for *E*_Bias_ = 2.5 V (occupied states). (e) Zoom of the region marked by the yellow rectangle in (b).

In this study, we focused on the surface deposition of heterobimetallic paddlewheel complex [PtVO­(SOCPh)_4_] in Figure 1a,^24^ **PtVO** hereafter, containing monothiobenzoate ligands. **PtVO** was deposited on highly oriented pyrolytic graphite (HOPG) using electrospray deposition (ESD) in ultra high vacuum (UHV), a soft processing method,^26,27^ with the dual purpose of assessing the applicability of ESD to complexes of this class and elucidating their organization and interfacial interactions on the graphite surface. To address these questions, we employed a combination of surface-sensitive characterization techniques, such as X-ray photoelectron spectroscopy (XPS), scanning tunneling microscopy (STM), and X-ray absorption spectroscopy (XAS). The results demonstrated that ESD enables the controlled formation of a submonolayer of **PtVO** on HOPG (hereafter **PtVO-ML**), while preserving both the molecular structure and the intrinsic magnetic properties of the complex. Furthermore, density functional theory (DFT) calculations were used to establish the most stable adsorption geometry, confirming that the electronic structure is fully preserved on the surface, and rationalizing the STM data. Polarization-dependent XAS, X-ray magnetic circular dichroism (XMCD), and X-ray natural linear dichroism (XNLD) spectra were reproduced by multiplet ligand field (MLF) theory simulations that explicitly include experimental geometry and polarization. Orbital resolved analysis further unveiled the microscopic origin of the angular dichroism and provided a direct link between angular dependent spectral features and the contribution of each 3d orbital, thereby establishing a robust spectroscopic fingerprint of molecular orientation.

Our findings establish the viability of vanadyl-based heterobimetallic lantern complexes as quantum building blocks on surfaces, bridging molecular design with interfacial engineering. These advances lay the groundwork for designing molecular architectures that simultaneously ensure quantum functionality, electronic robustness, and structural adaptability, paving the way toward their individual addressing and their integration into next-generation quantum devices.

## Experimental Section

### Electrospray Deposition

ESD was carried out using the commercial system MolecularSpray UHV 4i. A solution of **PtVO** (0.1 mM) in dry acetonitrile/CH_2_Cl_2_ (2:1 v/v) was pumped with a syringe pumping system at a rate of 0.5 mL/h through an emitter with an internal diameter of 100 μm. A bias of 2.7 kV was applied between the solution and the entrance capillary. Tests performed via XPS and STM characterization suggested that 20 min of spraying are required to obtain a submonolayer deposit. During the deposition, the substrate was kept at room temperature.

### XPS Measurements

XPS measurements were performed using a microfocused monochromatic Al Kα radiation source (1486.6 eV), SPECS XR-MS focus 600 operating at a power of 100 W (13 kV and 7.7 mA) and a SPECS Phoibos 150 1DLD electron analyzer with a pass energy of 40 eV to ensure an appropriate resolution. The spectra were acquired in normal emission with the X-ray source mounted at 54.44° with respect to the analyzer. The spectra were calibrated by rescaling the binding energy values to the C1s peak at 284.5 eV.^28^ Fitting analysis was performed using CasaXPS software^29^ introducing mixed Gaussian and Lorentzian contributions for each component. The background was fitted using the Shirley or linear method, depending on the nature of the background. A bulk reference sample was prepared by drop-casting the same solution used for the ESD. The adopted semiquantitative analysis was performed by employing the calculated cross-section values extracted from the literature.^30^

### STM Measurements

The STM measurements were carried out using an Omicron variable-temperature VT-SPM setup operated in vacuum and a Pt/Ir mechanically prepared tip. The STM images were acquired at 35 K in order to suppress molecular diffusion on the surface and ensure positional stability of the adsorbed molecules during imaging.

### Computational Methods

The CP2K 8.2 quantum chemistry software^31^ was employed for all the periodic DFT (pDFT) calculations. RevPBE functional,^32,33^ along with rVV10 empirical dispersion corrections,^34^ were used in all geometry optimizations. The adsorption geometries were evaluated at the Γ point. Norm-conserving Goedecker–Tetter–Hutter pseudopotentials^35^ and a double-ζ basis set with polarization functions (DZVP-MOLOPT-SR) were employed for all the atoms. The unit cell parameters were kept fixed throughout the optimizations. The plane-wave cutoff value was set to 450 Ry. The wave function convergence threshold (EPS_SCF) was set to 1.0 × 10^–6^ Hartree, while the max force for the geometry optimization was set to 4.5 × 10^–3^ bohr^–1^ Hartree. A single **PtVO** molecule adsorbed on a 13 × 13 graphene layer was simulated in three different adsorption configurations (see Figure S1). A hexagonal unit cell (32.1035 Å × 32.1035 Å × 40.0000 Å, α = β = 90°, γ = 60°) was used throughout these optimizations. In all three configurations, all atomic positions were allowed to relax. The unit cell size was chosen to avoid interaction between replicas of the adsorbed **PtVO**. To compute the adsorption energy, an isolated molecule of **PtVO** and the graphene surface were separately optimized within the same unit cell. The adsorption energy (*E*_ads_) was then evaluated with the formula *E*_ads_ = *E*_mol@surf_ – (*E*_mol_ + *E*_surf_), where the three terms are the computed electronic energies of the molecule adsorbed on graphene (*E*_mol@surf_), of the isolated molecule (*E*_mol_), and of the bare graphene surface (*E*_surf_) in their optimized geometries.

### XAS Measurements

XAS experiments were performed at the DEIMOS beamline^36^ (SOLEIL synchrotron, France), employing both linear and circular polarizations and using total electron yield detection^37^ to achieve surface sensitivity. Samples were transported to the DEIMOS beamline using an ultrahigh vacuum (UHV) suitcase (*P*_base_ = 5 × 10^–10^ mbar). All XAS data were acquired at 2 K under an applied magnetic field of ±6 T collinear with the X-ray propagation vector (***k***). The spectra were recorded at the V L_2,3_ edges at specific values of the angle (θ) between ***k*** and the surface normal.

### XAS Simulations

The polarized X-ray absorption spectra were simulated using the script language Quanty,^38^ within the MLF framework.^39^ A custom script was developed to incorporate the exact experimental geometry and polarization of the incident light. Temperature effects were accounted for by weighting the transition probabilities according to the Boltzmann population of the 2p^6^3d^1^ ground electronic configuration of the vanadyl ion. To reproduce the experimental spectra, an intrinsic Lorentzian line width of 70 meV was included. Additionally, an energy-dependent Gaussian broadening was added to capture the varying line widths at the L_2,3_ edges.

## Results and Discussion

The synthesis of the monothiobenzoato-bridged lantern complex **PtVO** (Figure 1a) in pure crystalline form was carried out following previously reported procedures.^24^ Further details on the synthetic route and solution characterization data are provided in Supporting Information Note S1. A submonolayer of **PtVO** was deposited by ESD onto HOPG, selected as a chemically inert substrate to minimize molecule–substrate interactions^4,40–43^ and to suppress chemisorption, which is instead typically observed on coinage metal surfaces. This approach has proven effective for transferring large and nonvolatile molecules onto surfaces while minimizing fragmentation.^44–46^ A highly diluted solution of **PtVO** in an acetonitrile/CH_2_Cl_2_ mixture was sprayed through sequential pumping stages onto a HOPG substrate under UHV conditions.

The resulting submonolayer was initially characterized by STM to gather insights into the molecular organization on the surface (Figure 1b). The resolution of the STM images is limited at the lowest temperature accessible in our setup (35 K), indicating that molecules interact only weakly with the HOPG substrate and maintain significant mobility. The images revealed isolated bright round features with a size of about 2 nm ascribed to individual molecules (Figures 1b,e and S2), while long-range ordered packing was not observed.

pDFT calculations were performed to explore three possible **PtVO** orientations on graphene: standing with the pseudo-*C_4_* axis perpendicular to the surface and the vanadyl oxygen pointing toward the vacuum, lying with the *C_4_* axis almost parallel to the graphene substrate, and upside-down with the vanadyl oxygen pointing toward the surface (Figures 1d and S1a–c). The computed adsorption energies (see Table S1) clearly indicate that **PtVO** preferentially interacts with graphene through the sulfur atoms in the standing configuration (Figure 1c). The computed STM images of isolated molecules in the three configurations (Figure S1d–f) exhibit distinct dimensions and intensity profiles (Figure S1g–i). Comparison with the experimental images and their corresponding profiles (Figure S2) indicates that the observed features are consistent with either a standing or an upside-down configuration, while the lying configuration can be excluded. No chemisorption is expected, as all computed adsorption energies are below 30 kcal/mol. Charge and spin population analyses (Table S2) further support the absence of significant electron transfer between the molecule and the surface. Overall, the results indicate that molecular adsorption is primarily governed by van der Waals interactions, which preserve molecular structure but hinder the formation of stable, ordered layers.

According to the calculations, the singly occupied molecular orbital (SOMO) is the d_*xy*_ orbital localized on vanadium (Figure 4c), as expected for a vanadyl complex in a square planar coordination environment. Consistent with previous experimental and theoretical work on the bulk phase,^24^ the orbital shows a minor degree of delocalization onto platinum mainly through the monothiocarboxylate ligands. This through-bond mechanism leads to a spin density on Pt comparable to that found on the oxygen atoms directly bonded to the vanadium ion. This behavior remains substantially unchanged upon moving from the isolated molecule to the adsorbed phase (see Table S2).

The deposited sample was further analyzed by XPS to verify the stability of the molecular structure upon deposition. For comparison, a reference bulk-like sample was prepared by drop-casting molecules from the same solution used for the ESD. The spectral analysis focused on the S2p, Pt4f, and V2p regions, which serve as sensitive probes for chemical modification. In the S2p region (Figure 2a), both bulk and deposited samples exhibit a single component at 162.9 eV, accompanied by its spin–orbit coupled one, consistent with the four equivalent sulfur atoms in the monothiocarboxylate ligands. The spectra confirm that the sulfur environment remains unperturbed upon surface deposition and that the interaction between the molecule and the substrate does not significantly alter the electronic state of sulfur. A similar outcome is observed in the Pt4f region (Figure 2b), where both samples show a strong single Pt4f_7/2_ component (73.1 eV for the bulk, 73.0 eV for the submonolayer), corresponding to the unique chemical environment of platinum in the **PtVO** complex. The negligible spectral differences between bulk and deposited samples indicate that the platinum center retains its oxidation state and coordination geometry. Crucially, the V2p region (Figure 2c) displays a single V2p_3/2_ component at 517.0 eV for the bulk and 516.8 eV for the submonolayer, attributable to the vanadium atom in the vanadyl unit. In this region, both bulk and submonolayer deposits exhibit an additional peak at 520.8 eV, which is, however, due to the Pt4p_3/2_ signal. Note that XPS experiments on vanadyl phthalocyanines and porphyrins on different surfaces evidenced two distinct V2p components due to the presence of different molecular orientations of the vanadyl group (standing and upside-down).^9,10,47^ In the present case, however, the XPS spectra suggest a uniform molecular alignment on the HOPG surface, consistent with theoretical calculations, and exclude an upside-down configuration that would generate an additional contribution at lower binding energies due to screening effects.^10^ The elemental composition estimated from XPS signal integration (Table S3) agrees with the stoichiometry of the pristine material, confirming the success of the ESD method.

**Figure 2:**
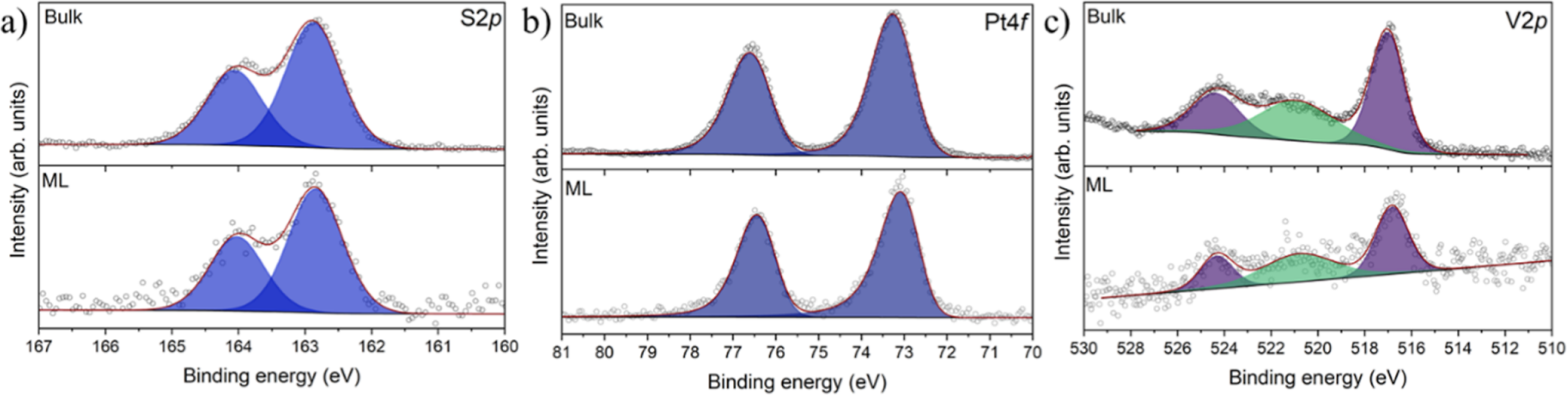
(a) S2p, (b) Pt4f, and (c) V2p XPS spectra of **PtVO** in the bulk phase and **PtVO-ML** on HOPG.

The adsorption geometry of **PtVO** was also investigated by synchrotron-based XNLD measurements performed at the vanadium L_2,3_ edges (Figure 3a). The XNLD spectrum, obtained as the difference of the XAS signals recorded with vertical (σ^*V*^) and horizontal (σ^*H*^) linear polarization and a consequent background correction (see Supporting Information Note S2), reveals a strong dichroic response (ca. 40%), which is consistent with a preferential standing adsorption geometry of **PtVO** on the HOPG substrate. This spectroscopic fingerprint, in combination with XPS measurements, DFT results, and the multiplet analysis described below, converges toward a coherent picture of a preferential standing adsorption geometry. While the overall dichroic profile resembles that reported for vanadyl porphyrins and phthalocyanines,^4,10^ the distinct feature at 518.6 eV appears to be more specific to this lantern-type compound. The additional dichroic features observed above 530 eV can be assigned to the K-edge of oxygen. Their origin can be traced back to the presence of oxygen directly coordinated to the vanadium moiety, which is visible due to the in situ nature of the sample and the absence of spurious oxygen contamination. The molecular adsorption geometry was definitively established using MLF calculations described later in the text.

**Figure 3:**
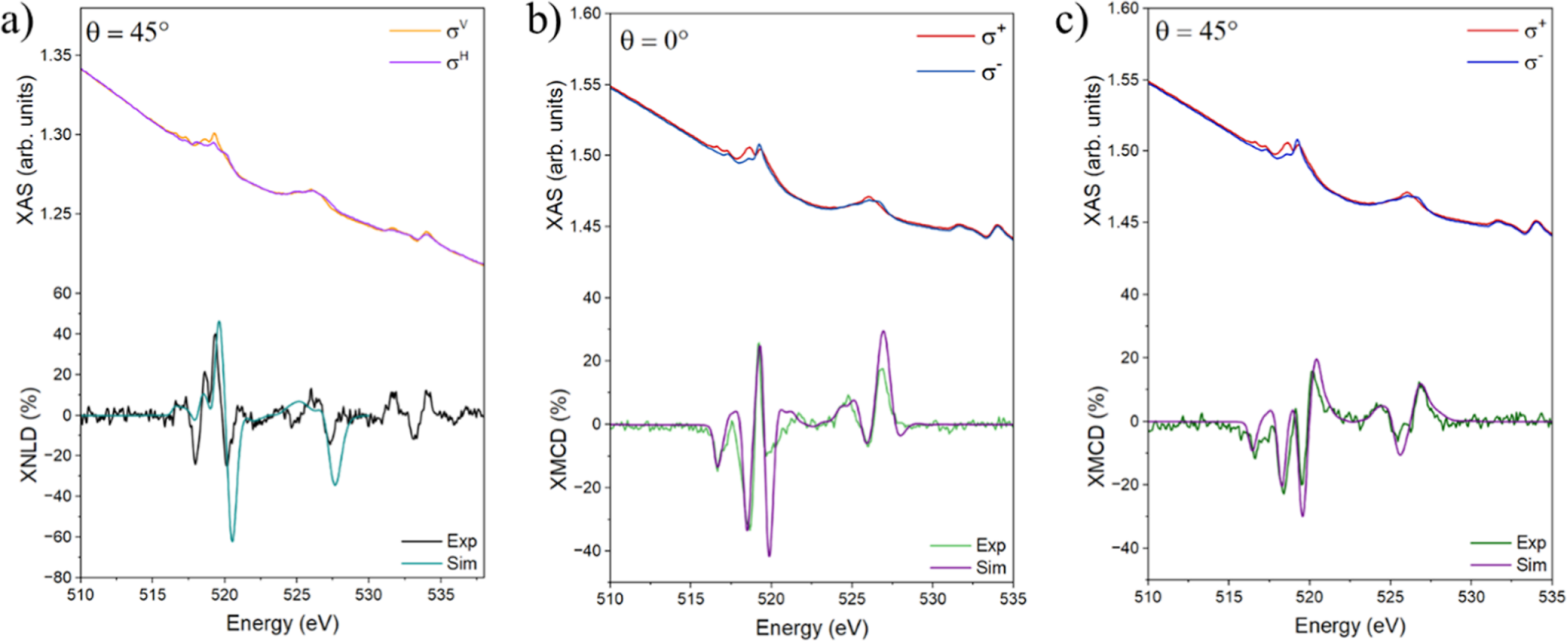
(a) Simulated and experimental vanadium L_2,3_ XNLD spectra of **PtVO-ML** at θ = 45°. Experimental data include signal arising from oxygen K-edge. (b) Simulated and experimental vanadium L_2,3_ simulated and experimental XMCD spectra of **PtVO-ML** at θ = 0°. (c) Simulated and experimental vanadium L_2,3_ XMCD spectra of **PtVO-ML** at θ = 45°. The corresponding XAS traces are provided in the upper part of the diagrams. All the spectra were acquired at *B* = 6 T and *T* = 2 K.

Circularly polarized X-ray absorption experiments were used to probe the magnetic properties of the adsorbed species. The XMCD profile was obtained as the difference between the signals recorded with right (σ^–^) and left (σ^+^) circularly polarized light. At normal incidence (θ = 0°, Figure 3b), the XMCD spectrum displays a main dichroic peak at 518.6 eV, with a line-shape comparable to that reported for other vanadyl-based compounds.^4,9,10^ To validate this assignment, the spectrum was compared to that of a drop-cast **PtVO** sample on HOPG, which serves as a bulk-like reference (Figure S3). The good agreement between the two data sets confirms that the vanadyl ion preserves its localized spin at the V center upon adsorption. To gain insight into the magnetic anisotropy of the submonolayer deposit, XMCD spectra were also recorded at θ = 45° (Figure 3c). Clear changes in the XMCD intensity were observed, consistent with previous reports on similar molecular frameworks.^48^ In addition, in the spectra acquired at θ = 45°, an extra feature emerges at 520.2 eV, which is characteristic of vanadyl systems.^4^

To rationalize these experimental observations, the polarized X-ray absorption spectra were simulated using the Quanty program.^49^ A custom script was developed to account for the exact experimental conditions used, including the polarization of the incident beam. The spectra were obtained as the weighted sum of the transition probabilities between the eigenstates of the ground (2p^6^3d^1^) and excited (2p^5^3d^2^) electronic configurations of the vanadium­(IV) center. The eigenstates were obtained by diagonalizing the many-body Hamiltonian *Ĥ* reported in eq 1.

1 $$Ĥ={Ĥ}_{ee}+{Ĥ}_{SOC}+{Ĥ}_{CF}+{Ĥ}_{Zeeman}$$

Here, the individual terms represent interelectronic repulsion, spin–orbit coupling (SOC), crystal field (CF), and Zeeman interactions, respectively. For the ground configuration, which contains a single 3d electron, the interelectronic repulsion term can be neglected. In contrast, in the excited configuration, the presence of open 2p and 3d shells requires both direct (*dd*) and exchange (*pd*) Coulomb interactions to be included. These parameters were taken from the literature^50^ and uniformly scaled by an empirical reduction factor κ = 0.8. The SOC constants for both 3d and 2p shells were also fixed to literature values.^50^ A full list of the employed single-ion parameters is provided in Table S4.

For an initial guess of the CF interaction, we performed CASSCF calculations, which yielded the energy structure for the 3d^1^ electronic configuration reported in Table S5. The results are consistent with a square pyramidal coordination environment, in which the electronic states derived from the 3d^1^ configuration transform as the *B*_1_ (*d*_*x^2^*__–*y^2^*_), *B*_2_ (*d*_*xy*_), *E* (*d*_*xz*_,*d*_*yz*_) and *A*_1_ (*d*_*z^2^*_) irreducible representations. From ab initio calculations, the electronic eigenstates of *B*_2_, *B*_1_, *E* and *A*_1_ symmetry lie at 0, 2.0452, 2.4758, and 4.3984 eV, respectively. It should be noted here that CASSCF does not yield perfectly degenerate d_*xz*_ and d_*yz*_ orbitals. Therefore, the value for the *E* irreducible representation was taken as the average energy of these two orbitals. Moreover, according to CASSCF calculations, the d_*xz*_ and d_*yz*_ orbitals are higher in energy than the $d_{x^{2}-y^{2}}$ orbital, a trend also observed in other square pyramidal complexes^51^ and attributable to the competition between equatorial and axial CF strengths. Furthermore, we emphasize that the CASSCF calculations, performed on the extrapolated adsorbed geometry, yield an electronic structure that is in full agreement with the results of the calculations performed on the crystallographic and gas-phase structures.^24^ The CF Hamiltonian was parametrized accordingly, as described in Supporting Information Note S3, and assumed to be identical in the ground and excited electronic configurations. However, simulations performed with these initial parameters showed poor agreement with the experimental spectra (Figure S4), underscoring the need to refine the Hamiltonian parameters by fitting the experimental XMCD and XNLD spectra.

A straightforward adjustment of the CF parameters alone failed to yield significantly improved simulations, indicating that additional contributions must be considered. Following previous reports on vanadyl-based molecular systems,^48,52^ the empirical reduction factors for both the *dd* and *pd* Coulomb interactions (denoted as κ_dd_ and κ_pd_, respectively) were also left free to vary in the fit. This procedure yielded a markedly improved agreement with experiments (Figure 3), with best-fit parameters 0 eV (*B*_2_), 1.44 eV (*B*_1_), 2.00 eV (*E*) and 3.38 eV (*A*_1_), κ_dd_ = 0.474 and κ_pd_ = 0.535.

A few considerations can be drawn from these results. First, in earlier studies, a strong reduction of the interelectronic repulsion parameter was also shown to be essential for reproducing the experimental XAS profiles of vanadyl-based systems.^48,52^ This reduction is commonly attributed to the covalent character of the vanadyl-ligand interaction,^53,54^ which effectively screens the 3d electrons and decreases the effective Coulomb repulsion. In ligand-field theory, this is often referred to as the nephelauxetic effect.^55^ However, the magnitude of the reduction observed here indicates that a purely ionic ligand field model is inadequate, and that an explicit treatment of covalency is required. In principle, this can be achieved within Quanty by including ligand-to-metal charge transfer (LMCT) configurations, in which ligand orbitals hybridize with metal orbitals. Additionally, while the simulated dichroic features are located at the right energies, discrepancies remain in the intensity of the signals. This indicates that the overall excited-state energy structure is correctly captured, but the transition probabilities, which are linked to the orbital composition of the states, are not. Explicitly including covalency would provide a more realistic description of the electronic structure and could, in principle, modify the orbital compositions and improve the dichroic intensities. Yet, such an approach would also dramatically increase the Hilbert space, making a direct fitting of the experimental data computationally highly demanding.

A second important point concerns the CF interaction. The best-fit parameters obtained from the XAS simulations deviate significantly from those obtained from both ab initio calculations and UV–vis measurements (Table S6).^25^ Nonetheless, this discrepancy requires careful interpretation. In XAS simulations, thermal effects are included by weighting transition probabilities with the Boltzmann population of the electronic ground state levels. For vanadyl ions, the 3d^1^ configuration is strongly split, with the excited doublets lying well above *k*_*B*_ *T* and being essentially unpopulated (see Table S5). Consequently, all XAS transitions originate exclusively from the ground doublet, making the simulated XAS spectra insensitive to the CF parameters of the 3d^1^ configuration. On the contrary, the XAS spectra should be sensitive to the CF interactions in the 2p^5^3d^2^ final state. This is confirmed by simulations: when the ground state is described with CF parameters derived from UV–vis spectroscopy and the excited state with the best-fit parameters, the calculated spectra remain unvaried. This observation is notable, as it suggests that while UV–vis spectroscopy is an important probe for the CF splitting in the 3d^1^ configuration, XAS at the L_2,3_ edges can directly probe the CF interaction in the 2p^5^3d^2^ configuration. The additional 2p core hole in the excited state modifies the electronic potential sensed by the 3d orbitals and thus can lead to an effectively different CF splitting.^56^ A rigorous quantification of the excited state CF is, in general, hampered by the presence of many strongly correlated parameters. To avoid overparameterization, it is therefore commonly assumed that CF parameters are identical in the ground and excited states. By contrast, the simplicity of the 3d^1^ ground state configuration of vanadyl ions makes them a rare model system for the determination of the CF splitting in the 2p^5^3d^2^ excited state. In summary, the empirical reduction of the Coulomb repulsion parameters reflects strong metal–ligand covalency through the nephelauxetic effect, while the deviation of the CF parameters from the ab initio estimates is attributable to the modified electronic potential in the 2p^5^3d^2^ excited state. To better understand the origin of the dichroic features observed in the angular dependent XMCD spectra, we analyzed the wave functions and extracted the occupancy of the real 3d orbitals in the excited 2p^5^3d^2^ configuration. A similar approach was previously applied, from an ab initio perspective, to different oxidation states of vanadium.^57,58^ Based on the occupancies, the simulated spectra were reconstructed by weighting the contribution of each 3d orbital with the transition probabilities, which depend on the light polarization. A Lorentzian broadening was also introduced to account for the finite lifetime of the states, together with an energy-dependent Gaussian broadening, as in the Quanty script. A detailed description of the protocol is given in Supporting Information Note S4. Figure 4a,b shows the computed XMCD spectra (black lines) decomposed into the contributions of each 3d orbital (colored areas). As expected from the strong mixing of excited states, all orbitals contribute to each transition. However, the evolution of their importance across the L_3_ edge clearly reflects the CF ordering. The first dichroic peak, lying at the lowest energy, arises primarily from the 3d_*xy*_ orbital, followed by features dominated by $3d_{x^{2}-y^{2}}$ , 3d_*xz*/*yz*_ and finally 3d_*z*_^2^. Notably, the dichroic feature just below 520 eV is mainly associated with the $3d_{x^{2}-y^{2}}$ orbital and its intensity decreases when going from 0° to 45°, whereas the feature just above 520 eV, dominated by 3d_*xz*/*yz*_ and 3d_*z^2^*_ orbitals, shows opposite behavior, increasing with incidence angle. Simulations performed across the angular range 0°–60° confirm these trends (Figure 4d). A similar behavior is also observed at the L_2_ edge, although the broader line width at this edge^59^ may hinder quantitative analysis.

**Figure 4:**
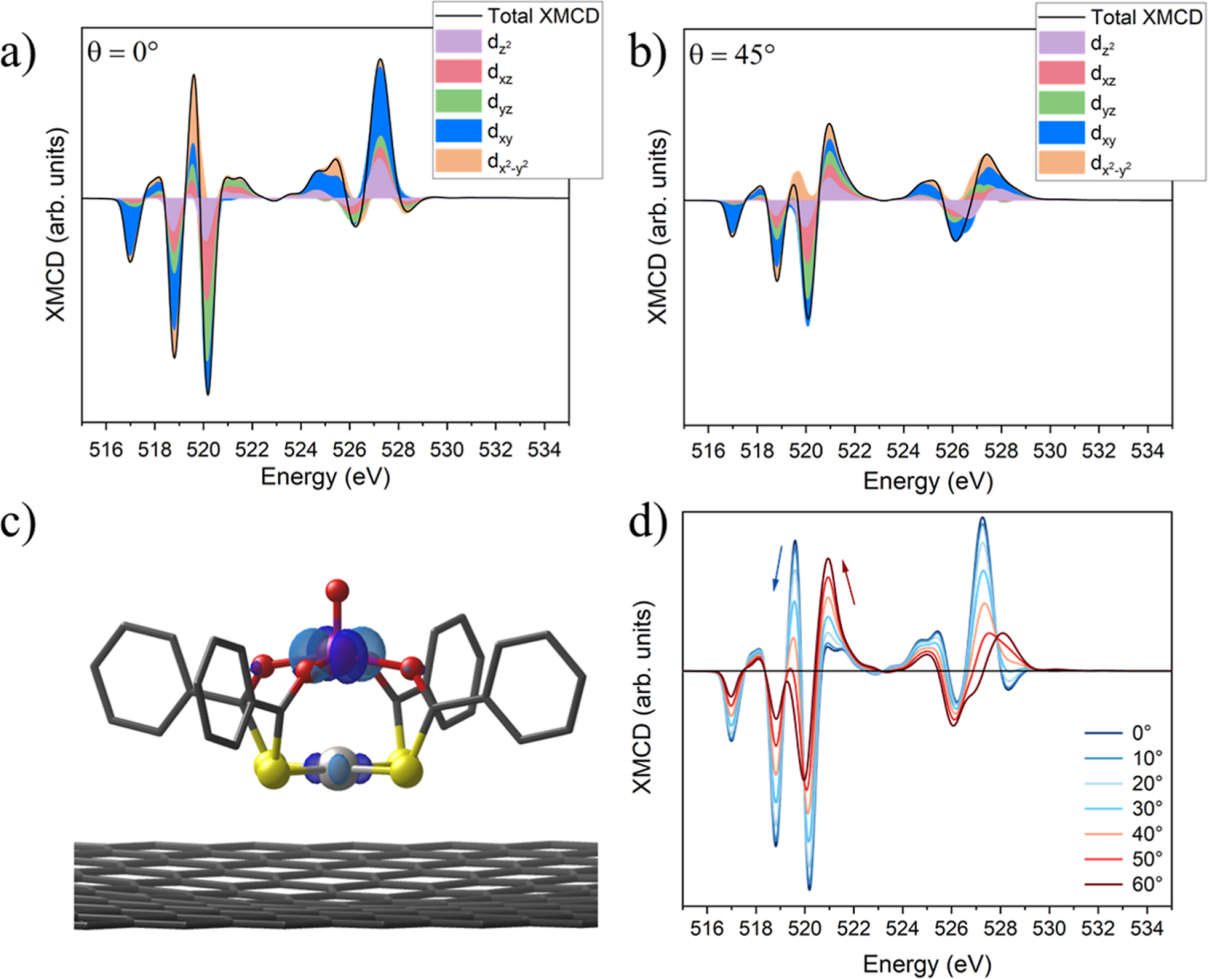
(a,b) Orbital-resolved decomposition of the XMCD spectra at θ = 0° and θ = 45° computed from the occupancy of the 3d orbitals in the excited electronic configuration 2p^5^3d^2^. The contribution of each 3d orbital is shown individually and does not overlap. The black lines represent the total computed XMCD spectra. (c) DFT simulated SOMO of **PtVO** in its standing adsorption geometry, plotted with isosurface level = 0.067 e^–^/a_0_^3^. (d) Angular dependence of the simulated XMCD spectra for the vanadyl ion from normal incidence (θ = 0°) to grazing incidence (θ = 60°).

Beyond rationalizing the individual dichroic features, the orbital-resolved decomposition reveals a clear structural-spectroscopic correspondence. The redistribution of intensity between in-plane and out-of-plane 3d contributions across the 520 eV region provides information regarding the microscopic origin of the angular dependence. Below 520 eV, the dichroism is characterized by in-plane-like 3d orbitals, whose weight is maximal at normal incidence and decreases as the beam moves away from the vanadyl axis. Above 520 eV, the trend is reversed: the predominantly axial orbitals contribute weakly at 0° but generate strong dichroism once the beam acquires an in-plane component. This inversion, unlike the lower-energy L_3_ features whose phase is insensitive to angle, provides a clear electronic structure rationale for the angular evolution of the spectra. Importantly, this behavior does not arise solely from the spatial orientation of individual 3d orbitals. Instead, the decomposition reflects how different CF manifolds, strongly mixed in the 2p^5^3d^2^ final states, influence distinct energy regions and respond differently to the experimental geometry. The change in their relative weight across the 520 eV threshold correlates with the observed angular response (Figure 3b,c), yielding a microscopic explanation for the dichroic inversion and offering a practical spectral fingerprint for assessing the molecular orientation of square-pyramidal vanadyl complexes, even if their magnetic response is practically isotropic.

## Conclusion

In this work, we unveiled the structural and magnetic integrity of a submonolayer of **PtVO** on HOPG, demonstrating the suitability of these heterobimetallic paddlewheels for surface deposition by ESD, and thus identifying this system as a promising platform for single spin addressing by local spectroscopies. XPS analysis confirmed the preservation of the molecular structure upon processing, while DFT calculations revealed that the most stable adsorption geometry involves a standing orientation of molecules, with the sulfur atoms of the monothiobenzoate ligands in contact with the HOPG surface. Importantly, the theoretical results indicated the absence of spin delocalization onto the substrate, ensuring the magnetic isolation of the paramagnetic center. Synchrotron-based XMCD and XNLD measurements further substantiated the robustness of the molecular magnetic properties and the long-range structural orientation within the submonolayer. Simulations of polarization-dependent XAS spectra enabled the determination of the molecular orientation on the surface, which was found fully consistent with the DFT adsorption model. Moreover, the analysis revealed that only the CF splitting acting on the core-excited 2p^5^3d^2^ configuration significantly affects the XAS line shape, whereas the CF parameters in the ground state configuration play essentially no role. Finally, decomposition of the angular dependent XMCD spectra into the contributions of the individual 3d orbitals allowed to identify the microscopic origin of the dichroic features at the L_3_ edge and to rationalize their angular dependence, establishing angular-resolved XMCD as a sensitive probe of molecular orientation in vanadyl systems through orbital resolved excited state effects, even if the magnetic response of the system is essentially isotropic.

These findings prove that the unique molecular architecture of **PtVO** promotes a well-defined standing adsorption geometry and, at the same time, spatially and electronically decouples the paramagnetic metal center from the substrate. Beyond the inherent properties of the graphene substrate, the PtS_4_ platform acts as an additional inorganic buffer. By increasing the distance between the vanadyl unit and the surface, it suppresses hybridization and exchange coupling with the substrate’s itinerant electrons, which is crucial for maintaining quantum coherence. Therefore, it offers a novel molecular design paradigm for realizing controlled single-spin manipulation directly on surfaces, without relying on conventional inorganic decoupling layers. Furthermore, the delocalization of the spin density from vanadium to platinum, and the associated hyperfine coupling, could be leveraged to expand the Hilbert space available for quantum logical operations. Our work thus provides an important step toward the local single spin addressing on surfaces and its prospective integration into scalable spintronic and quantum devices.

## Supplementary Material



## Notes

pDFT relaxed geometries, STM dimension distributions, XMCD of bulk sample, XMCD simulation using CF parameters, calculated adsorption geometries, Loewdin population analysis, XPS semiquantitative analysis, parameters used for XAS simulations, synthetic procedure, XNLD raw data, reconstruction of XAS from 3d orbitals occupancy (PDF)

## #

CIC nanoGUNE, Tolosa Hiribidea 76, 20018, Donostia/San Sebastián, Spain

## ¶

School of Chemistry, Trinity Biomedical Sciences Institute, Trinity College Dublin, D02R590, Dublin, Ireland
