# Supplementary material for: Deposition of an Addressable Molecular Spin Qubit with Built-In Decoupling Structure
Source: J Am Chem Soc. 2026 May 7;148(19):19712–21. doi: 10.1021/jacs.6c01396 (PMC13195646; doi:10.1021/jacs.6c01396)
Supplement: Supplementary file 1 [file ja6c01396_si_001.pdf]

Supporting Information

Deposition of an addressable molecular spin qubit with built-in decoupling structure.

Niccolò Giaconi, [†1] *Leonardo Tacconi,* [†1] *Matteo Briganti,* [1] *Alessio Nicolini,*[2] *Olga Mironova,*[2] *Marta Albanesi,*[1,3] *Julie Lion,*[4] *Fabio Santanni,*[1] *Edwige Otero,*[3] *Philippe Ohresser,*[3] *Giulia Serrano,*[4] *Lorenzo Poggini,*[1,5] *Andrea Cornia,*[*2] *Matteo Mannini.* [*1]

[1] Department of Chemistry "Ugo Schiff" (DICUS), and INSTM Research Unit, University of Florence, Via della Lastruccia 3-13, 50019 Sesto Fiorentino, Italy.
[2] Department of Chemical and Geological Science and INSTM Research Unit, University of Modena and Reggio Emilia, Via G. Campi 103, 41125 Modena, Italy.
[3] Synchrotron-SOLEIL, L'Orme des Merisiers, 91190 Saint-Aubin, France.
[4] Department of Industrial Engineering (DIEF) and INSTM Research Unit, University of Florence, Via Santa Marta 3, 50139 Florence, Italy.
[5] Institute for Chemistry of Organo-Metallic Compounds (ICCOM-CNR), Via Madonna del Piano, 50019 Sesto Fiorentino, Italy.

[†] *These authors contributed equally to this work.*

Content

Figure S1. Relaxed geometries at the periodic Density Functional (pDFT) level of theory: a) *standing* , b) *lying* , and c) *upside-down.* Color code: C, grey; S, yellow; O, red. Simulated STM images of **PtVO** in d) *standing*, e) *lying*, and f) *upside-down* configurations. g-i) Profiles extracted from the simulated STM images along the corresponding green and red lines. 2

Figure S2. Lateral and vertical dimension distribution extracted from STM image reported in Figure 1e.. 3

Figure S3. XMCD spectra at the V $L_{2,3}$ edges acquired on a dropcast sample of **PtVO** on HOPG. The spectrum was acquired at B = 6 T and T = 2 K. 3

Figure S4. Comparison between experimental (black line) and simulated (red line) XMCD spectra acquired with an incident angle θ of 0° (a) and 45° (b). Simulations were performed using CF parameters obtained from *ab initio* calculations and using the interelectronic repulsion reduction factor $\kappa = 0.8$. 4

Table S1. Adsorption energies computed for **PtVO** over graphene by periodic density functional theory calculations, as described in main text. 5

Table S2. Loewdin population analyses for **PtVO** over graphene (standing/oxygen up configuration) and isolated molecule. For the adsorbed geometry, the total spin delocalization on the substrate is 0.0018 e^-... 5

Table S3. XPS semi-quantitative analysis estimated from V2p, S2p and Pt4f regions. 5

Table S4. Free-ion parameters used during XAS simulations. F and G represent the Slater-Condon integrals, while ζ is the spin-orbit coupling constant, referred to the orbitals as subscripts. The letter i and f refer to the initial state $2p63d1$ and final state $2p53d2$, respectively. Parameters are expressed in eV. 6

Table S5. Energy levels arising from the $3d^1$ configuration as determined from ab initio calculations....... 6

Table S6. Energy levels observed from UV-Vis measurements. 6

Note 1 – Synthetic procedure. 7

Note 2 – XNLD data treatment. 7
Note 3 – Crystal field parameters as a function of square pyramidal (C_{4v}) irreducible representations....... 8
Note 4 – Reconstruction of XAS spectra from 3d orbitals occupancy. 8

Supporting Figures

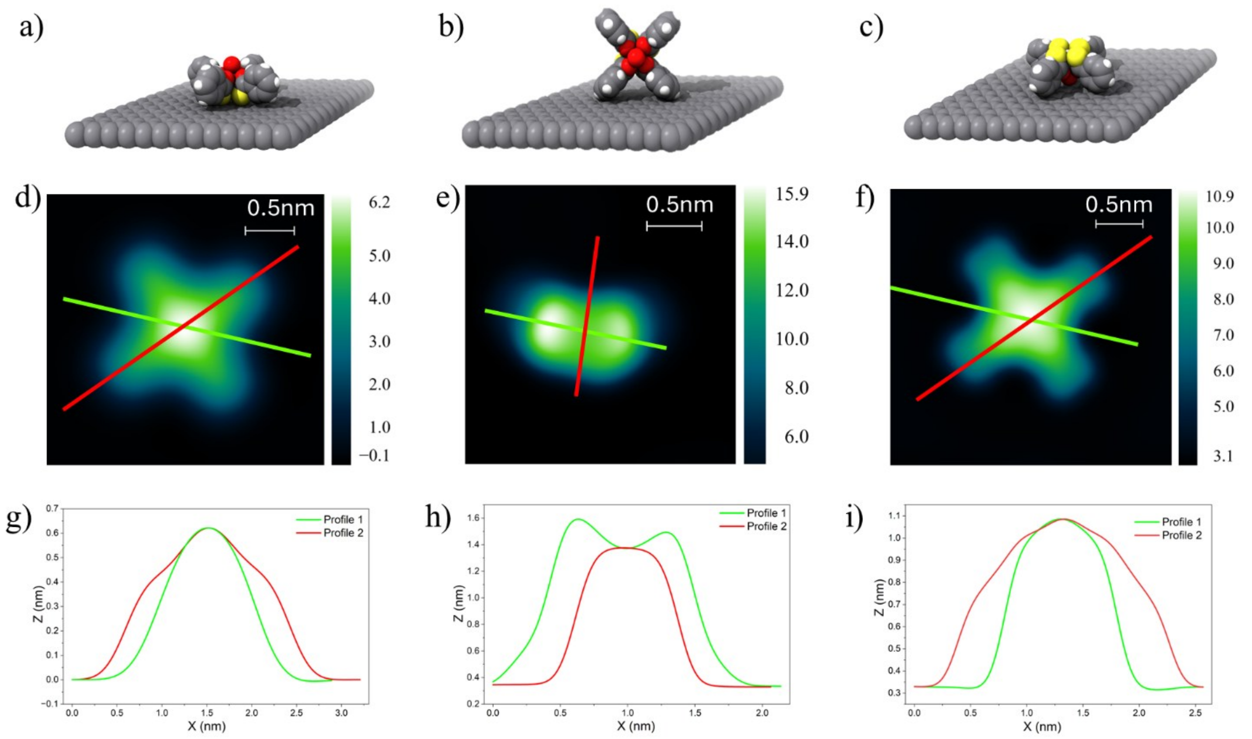

Figure S1. Relaxed geometries at the periodic Density Functional (pDFT) level of theory: a) *standing* , b) *lying* , and c) *upside-down.* Color code: C, grey; S, yellow; O, red. Simulated STM images of **PtVO** in d) *standing*, e) *lying*, and f) *upside-down* configurations. g-i) Profiles extracted from the simulated STM images along the corresponding green and red lines.

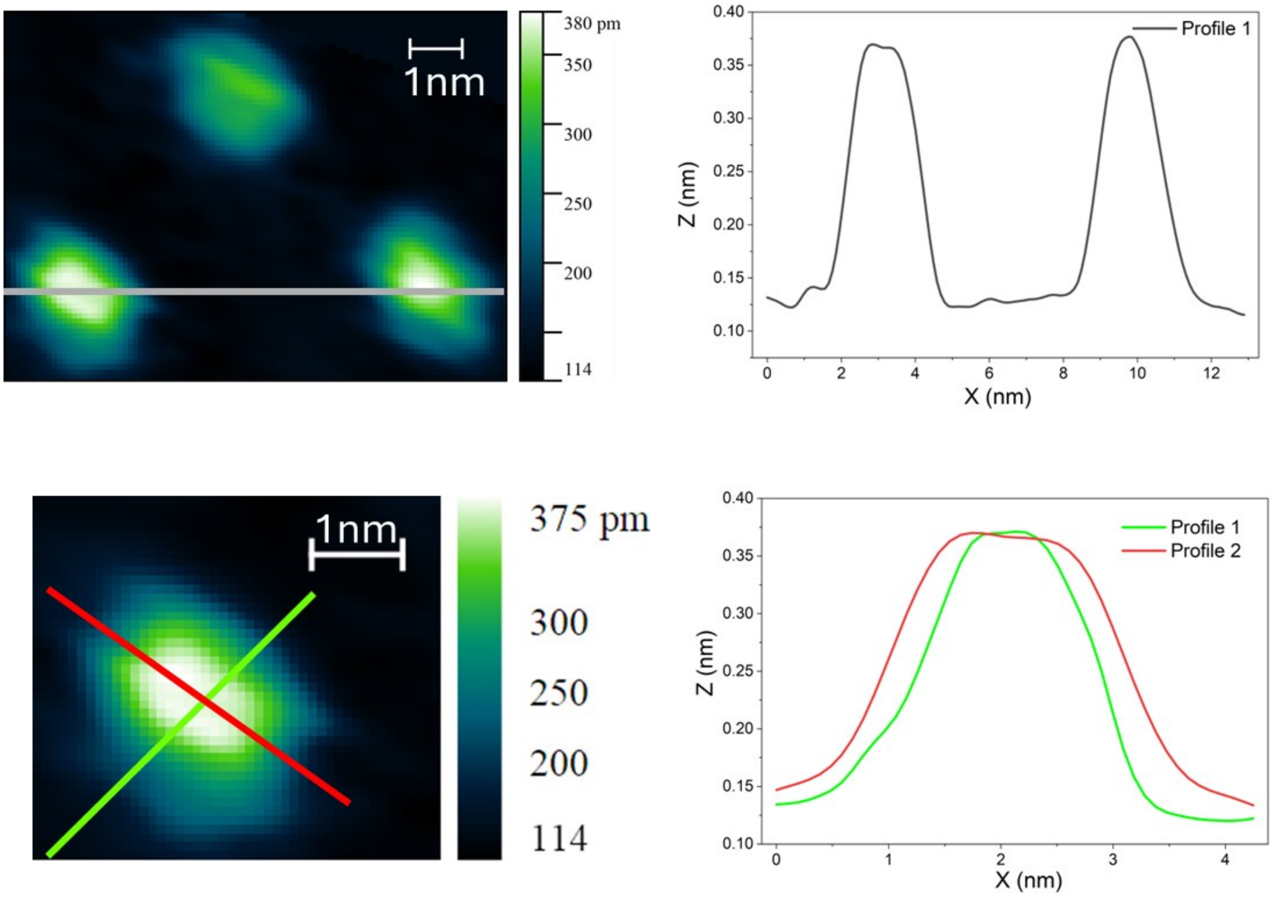

Figure S2. Lateral and vertical dimension distribution extracted from STM image reported in Figure 1e.

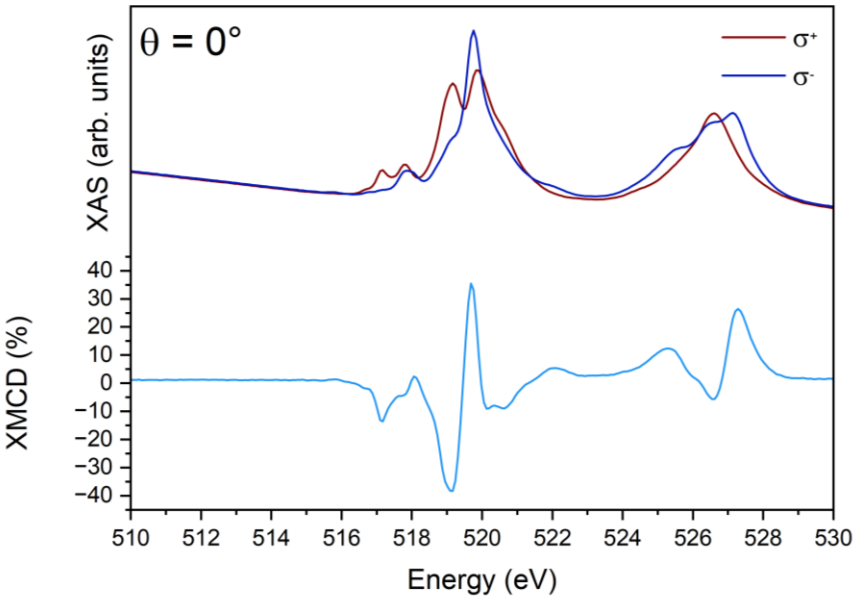

Figure S3. XMCD spectra at the V $L_{2,3}$ edges acquired on a dropcast sample of **PtVO** on HOPG. The spectrum was acquired at B = 6 T and T = 2 K.

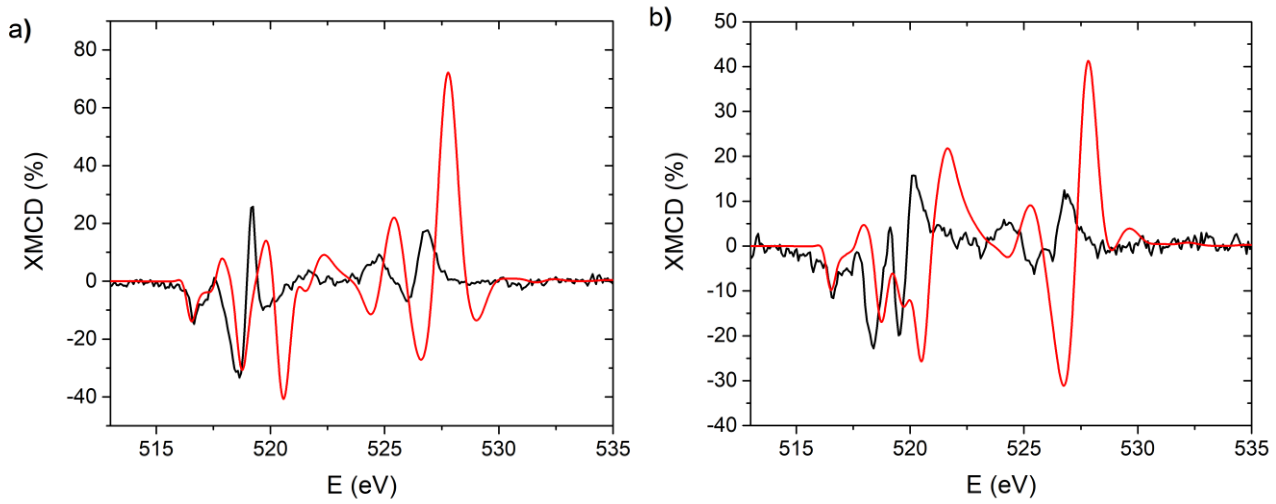

Figure S4. Comparison between experimental (black line) and simulated (red line) XMCD spectra acquired with an incident angle θ of 0° (a) and 45° (b). Simulations were performed using CF parameters obtained from *ab initio* calculations and using the interelectronic repulsion reduction factor $\kappa = 0.8$.

Supporting Tables

Table S1. Adsorption energies computed for **PtVO** over graphene by periodic density functional theory calculations, as described in main text.

**Configuration**	**Adsorption Energy (kcal/mol)**
Standing – oxygen up	-27.98
Upside-down – oxygen down	-21.25
Lying	-12.62

Table S2. Loewdin population analyses for **PtVO** over graphene (standing/oxygen up configuration) and isolated molecule. For the adsorbed geometry, the total spin delocalization on the substrate is 0.0018 e^-.

**Adsorbed on graphene (*Standing* – oxygen up)**			**Isolated molecule**		
Atom	Net charge	Spin moment	Atom	Net charge	Spin moment
Pt	-1.011071	0.029487	Pt	-1.012858	0.033780
V	0.362719	0.950982	V	0.366419	0.941257
S	0.749790	0.018306	S	0.714933	0.019913
S	0.746872	0.017301	S	0.716820	0.018538
S	0.743195	0.016509	S	0.716080	0.017926
S	0.750834	0.017031	S	0.715452	0.018260
O	-0.043672	-0.008124	O	-0.045167	-0.008305
O	-0.039939	-0.007529	O	-0.041335	-0.007926
O	-0.041886	-0.007835	O	-0.042742	-0.008319
O	-0.260213	-0.119708	O	-0.261503	-0.118044
O	-0.042057	-0.007588	O	-0.043280	-0.007922

Table S3. XPS semi-quantitative analysis estimated from V2*p*, S2*p* and Pt4*f* regions.

**Sample**	**V% (Theoretical)**	**S% (Theoretical)**	**Pt% (Theoretical)**	**Pt/V (Theoretical)**	**S/V (Theoretical)**
Bulk	18.5 ± 0.9 (16.7)	65.9 ± 3.3 (66.6)	15.6 ± 0.8 (16.7)	0.8 (1)	3.6 (4)
ESD	18.9 ± 0.9 (16.7)	61.2 ± 3.1 (66.6)	19.8 ± 1.0 (16.7)	1.0 (1)	3.2 (4)

Table S4. Free-ion parameters used during XAS simulations. F and G represent the Slater-Condon integrals, while ζ is the spin-orbit coupling constant, referred to the orbitals as subscripts. The letter i and f refer to the initial state $2p^6 3d^1$ and final state $2p^5 3d^2$, respectively. Parameters are expressed in eV.

$F^2(i)$	0	$F_d^2(f)$	11.9646
$F^4(i)$	0	$F_d^4(f)$	7.5540
$\zeta_d(i)$	0.031	$G_{pd}^1(f)$	5.012
		$G_{pd}^3(f)$	2.852
		$F_p^2(f)$	6.758
		$\zeta_d(f)$	0.042
		$\zeta_p(f)$	4.910

Table S5. Energy levels arising from the $3d^1$ configuration as determined from ab initio calculations.

Level	Energy (cm-1)	Energy (eV)	Boltzmann population at 300 K
|1>	0.00	0.00	0.500
|2>	0.00	0.00	0.500
|3>	16495.96	2.0452	0.000
|4>	16495.96	2.0452	0.000
|5>	19773.74	2.4516	0.000
|6>	19773.74	2.4516	0.000
|7>	20164.84	2.5001	0.000
|8>	20164.84	2.5001	0.000
|9>	35475.71	4.3984	0.000
|10>	35475.71	4.3984	0.000

Table S6. Energy levels observed from UV-Vis measurements.

Transition	Wavelength (nm)	Energy (eV)
1	439	2.88
2	620	2.00
3	729	1.79

Supplementary Notes

Note 1 – Synthetic procedure.

Complex **PtVO** was synthesized as previously reported.[1] Deuterated solvents were used as received. ^{1}H-NMR measurements were conducted at room temperature on an AVANCE400 FT-NMR spectrometer from Bruker Biospin (400.13 MHz), using 5 mm airtight Young-valved NMR tubes from Norell. The chemical shifts (δ) are expressed in ppm downfield from tetramethylsilane (TMS) as external standard.

Before electrospray deposition, the stability of **PtVO** in CD_3CN/CD_2Cl_2 (2:1 v/v) at 0.1 mM concentration was investigated *via* ^{1}H-NMR spectroscopy. Although the very broad signal of *m*-Ar protons was not visible due to the low concentration, the position of the broad singlet from *p*-Ar protons was similar to that observed in the reference spectra collected at higher concentrations (0.01 M and 0.2 mM) in pure CD_2Cl_2 ($\delta \sim 8.55$ vs. 8.50 ppm).[1] The spectrum remained unchanged over 24 hours, indicating high stability of **PtVO** in the solution used for electrospray deposition.

Note 2 – XNLD data treatment.

The XNLD spectrum was obtained as the difference between the XAS signals recorded with vertically and horizontally linearly polarized light. The corresponding raw data are shown in Figure S5.

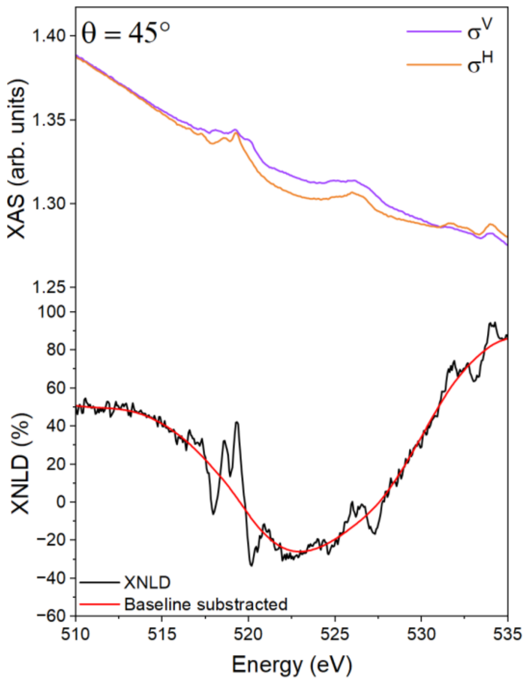

Figure S5 XAS and XNLD spectra at the V $L_{2,3}$ edges acquired on **PtVO-ML** sample before baseline correction. The spectrum was acquired at B = 6 T and T = 2 K.

In order to minimize the well-known spurious effects when dealing with tiny dichroic signals detected with linear polarisations produced by APPLEII undulator as well as with ordered substrates like HOPG, a background subtraction was applied to the XNLD spectrum by constraining the signal to zero in the pre-edge (515.75 eV) and the post-edge (521.35 eV and 530.0 eV) XNLD regions of the Vanadium L_3 edge. The background was then interpolated using a spline function and subtracted from the spectrum. Starting from the background-corrected XNLD spectrum, the corresponding XAS spectra reported in the main text (Figure 3a) were reconstructed using the following expressions, which account for the sample geometry with respect to the X-ray propagation vector **k**.

$$\sigma^V = ISO + \frac{XNLD}{3\,\cos^2\theta}$$

$$\sigma^H = ISO - \frac{(2\cos^2\theta - \sin^2\theta)\cdot XNLD}{3\cos^2\theta}$$

where $ISO = \frac{\sigma^V + \sigma^H}{2}$, *XNLD* denoted the background corrected dichroic spectrum, and θ is the angle (in radians) between the X-ray propagation vector **k** and the surface normal.

Note 3 – Crystal field parameters as a function of square pyramidal (C_{4v}) irreducible representations.

A square pyramidal crystal field split the $3d^1$ electronic configuration of a vanadyl ion into the four irreducible representations B_1, B_2, E and A_1. The square pyramidal crystal field Hamiltonian, expressed by the parameters B_0^0, B_0^2, B_0^4 and B_0^6, can be expressed as a function of the irreducible representations, as follows.

$$B_0^0 = \frac{1}{5}(A_1 + B_1 + B_2 + 2E)$$

$$B_0^2 = A_1 - B_1 - B_2 + E$$

$$B_0^4 = \frac{3}{10}(6A_1 + B_1 + B_2 - 8E)$$

$$B_4^4 = \frac{3}{2}\sqrt{\frac{7}{10}}(B_1 - B_2)$$

Note 4 – Reconstruction of XAS spectra from 3d orbitals occupancy.

In Quanty the wavefunctions of the investigated system are constructed starting from the spherical harmonics Y_l^m. In the case of the $3d$ shell, we have $l = 2$ and $m \in \{-2,-1,0,1,2\}$. The five real $3d$ orbitals, $d_{xy}, d_{yz}, d_{xz}, d_{z^2}, d_{x^2-y^2}$, are defined as linear combinations of spherical harmonics, as follows.

$$d_{z^2} \equiv Y_2^0$$

$$d_{yz} \equiv \frac{1}{\sqrt{2}}(Y_2^1 + Y_2^{-1}), \quad d_{xz} \equiv \frac{1}{\sqrt{2}}(Y_2^1 - Y_2^{-1})$$

$$d_{x^2-y^2} \equiv \frac{1}{\sqrt{2}}(Y_2^2 + Y_2^{-2}), \quad d_{xy} \equiv \frac{1}{\sqrt{2}}(Y_2^2 - Y_2^{-2})$$

In second quantization the occupancy of a spherical harmonic $|Y_l^m >$ in a many-body state $|\psi >$ can be calculated from the corresponding creation and annihilation operators, $a^\dagger_{Y_l^m}$ and $a_{Y_l^m}$, respectively.

$$n_{Y_l} = \langle\psi^* \left| a^\dagger_{Y_l^m} a_{Y_l^m} \right| \psi\,\rangle$$

The occupancy of any real $3d$ orbital can be derived in a similar manner. For example, the occupancy of the $3d_{xy}$ orbital can be calculated as

$$n_{d_{xy}} = \langle \psi^* \left| a^\dagger_{d_{xy}} a_{d_{xy}} \right| \psi \rangle$$

Because the operators transform under the same basis change as the orbitals, we obtain:

$$a^\dagger_{d_{xy}} = \frac{1}{\sqrt{2}} \left(a^\dagger_{Y_2^2} - a^\dagger_{Y_2^{-2}} \right)$$

$$a_{d_{xy}} = \frac{1}{\sqrt{2}} \left(a_{Y_2^2} - a_{Y_2^{-2}} \right)$$

From these Equation the occupancy can be further expanded as:

$$n_{d_{xy}} = \frac{1}{2} \langle \psi^* \left| a^\dagger_{d_{xy}} a_{d_{xy}} \right| \psi \rangle = \frac{1}{2} \langle \psi^* \left| a^\dagger_{Y_2^2} a_{Y_2^2} + a^\dagger_{Y_2^{-2}} a_{Y_2^{-2}} - a^\dagger_{Y_2^2} a_{Y_2^{-2}} - a^\dagger_{Y_2^{-2}} a_{Y_2^2} \right| \psi \rangle$$
$$= \frac{1}{2} (\rho_{22} + \rho_{\overline{2}\overline{2}} - \rho_{2\overline{2}} - \rho_{\overline{2}2})$$

Where the following relation stands:

$$\rho_{mn} = \langle \psi^* \left| a^\dagger_{Y^m_{|m|}} a_{Y^n_{|m|}} \right| \psi \rangle$$

Applying the basis transformation for the other real $3d$ orbitals, similar expressions can be derived.

$$n_{d_{z^2}} = \rho_{00}$$

$$n_{d_{yz}} = \frac{1}{2} (\rho_{11} + \rho_{\overline{1}\overline{1}} + \rho_{1\overline{1}} + \rho_{\overline{1}1})$$

$$n_{d_{xz}} = \frac{1}{2} (\rho_{11} + \rho_{\overline{1}\overline{1}} - \rho_{1\overline{1}} - \rho_{\overline{1}1})$$

$$n_{d_{x^2-y^2}} = \frac{1}{2} (\rho_{22} + \rho_{\overline{2}\overline{2}} + \rho_{2\overline{2}} + \rho_{\overline{2}2})$$

Therefore, extracting the eigenvectors of the system from the Hamiltonian it is possible to calculate the occupancy of each $3d$ real orbital in each eigenvector. Running these calculations on the electronic state $2p^6 3d^1$, delivers the following occupancy of the ten eigenstates $|n>$.

$$|1> \rightarrow n_{d_{xy}} = 1, \quad |2> \rightarrow n_{d_{xy}} = 1$$

$$|3> \rightarrow n_{d_{x^2-y^2}} = 1, \qquad |4> \rightarrow n_{d_{x^2-y^2}} = 1$$

$$|5> \rightarrow n_{d_{xz}} = 0.5, n_{d_{yz}} = 0.5, \quad |6> \rightarrow n_{d_{xz}} = 0.5, n_{d_{yz}} = 0.5$$

$$|7> \rightarrow n_{d_{xz}} = 0.5, n_{d_{yz}} = 0.5, \quad |8> \rightarrow n_{d_{xz}} = 0.5, n_{d_{yz}} = 0.5$$

$$|9> \rightarrow n_{d_{z^2}} = 1, \qquad |10> \rightarrow n_{d_{z^2}} = 1$$

These results confirm the ordering of the $3d$ orbitals based on the irreducible representations $B_2(d_{xy}) < B_1(d_{x^2-y^2}) < E\ (d_{xz}, d_{yz}) < A_1\ (d_{z^2})$.

The same calculations can be performed for each of the 270 states of the electronic excited state configuration $2p^5 3d^2$. Then, for each state $|\Psi(n) >$ the transition probability $P_j(n)$, can be calculated as follows

$$P_j(n) = \sum_{i=1}^{10} \left| \langle \Psi(n) \middle| \hat{O}(j) \middle| \psi(i) \rangle \right|^2 \cdot p_i(T)$$

where

$$p_i(T) = \frac{e^{-E_i/k_B T}}{Z(T)}, \quad Z(T) = \sum_{i=1}^{10} e^{-E_i/k_B T}$$

The operator $\hat{O}(j)$ is the dipole transition operator, which depends on the polarization j of the light. The full polarization dependent XAS spectrum $I_j(E,T)$ can then be computed as

$$I_j(E,T) = \sum_{n=1}^{270} P_j(n) \cdot B(E)$$

Where $B(E)$ is a broadening function composed by a constant Lorentzian contribution, having FWHM equal to 70 meV, and an energy dependent Gaussian contribution, which has a FWHM that linearly increases from 200 meV to 1 eV, in the 513 to 527 eV energy range.

References

1 M. Imperato, A. Nicolini, M. Borsari, M. Briganti, M. Chiesa, Y.-K. Liao, A. Ranieri, A. Raza, E. Salvadori, L. Sorace and A. Cornia, *Inorg. Chem. Front.*, 2024, **11**, 186–195.

2 G. van der Laan, *J. Phys. Condens. Matter*, 1998, **10**, 3239–3253.

3 P. Carra, B. T. Thole, M. Altarelli and X. Wang, *Phys. Rev. Lett.*, 1993, **70**, 694–697.

4 G. van der Laan, T. Hesjedal and P. Bencok, *Phys. Rev. B*, 2022, **106**, 214423.
